# Conocimientos de la hipertensión: Health beliefs about hypertension in an under-resourced community in the Dominican Republic

**DOI:** 10.1371/journal.pone.0235088

**Published:** 2020-06-23

**Authors:** Jasmine A. Abrams, Bryan Castro, Sushmita Gordhandas, Anna Grzegorczyk, Morgan Maxwell, Bridgette Brawner, Donaldson F. Conserve, Mark Ryan

**Affiliations:** 1 Department of Community Health Sciences, Boston University School of Public Health, Boston, Massachusetts, United States of America; 2 Center for Interdisciplinary Research on AIDS, Yale University School of Public Health, New Haven, Connecticut, United States of America; 3 Medical College of Virginia, Virginia Commonwealth University, Richmond, Virginia, United States of America; 4 Department of Obstetrics and Gynecology, University of Washington, Seattle, Washington, United States of America; 5 California Pacific Medical Center, San Francisco, California, United States of America; 6 Department of Population Health Sciences, Virginia Polytechnic Institute and State University, Blacksburg, Virginia, United States of America; 7 Department of Family and Community Health, University of Pennsylvania School of Nursing, Philadelphia, Pennsylvania, United States of America; 8 Department of Health Promotion, Education, and Behavior, Arnold School of Public Health, Columbia, South Carolina, United States of America; 9 Department of Family Medicine and Population Health, Virginia Commonwealth University, Richmond, Virginia, United States of America; Children's Mercy Hospitals and Clinics Department of Pathology and Laboratory Medicine, UNITED STATES

## Abstract

Understanding health beliefs is important to facilitate health promotion and disease prevention as they influence health behaviors, outcomes, and disease management. Given the rise of hypertension-related diseases in the Dominican Republic, the purpose of our study was to identify hypertension-related health beliefs of Dominicans in order to inform the development of culturally appropriate interventions for hypertension prevention, care, and treatment. Semi-structured interviews were conducted with 20 Dominicans, 15 of whom were receiving treatment for hypertension. Operating within the interpretative paradigmatic framework, we conducted thematic analyses of interview data to identify hypertension-related health beliefs and practices. Iterative data analysis revealed the following themes: 1) Negative emotions are a primary cause of hypertension, 2) Medication is the best treatment but adherence is challenging, 3) Systemic barriers impede treatment access, 4) Hypertension negatively impacts mental and physical well-being, and 5) Lifestyle changes, relaxation, and social support help manage hypertension. Data gathered from member checking validated these findings. This study enhances understanding of the beliefs and experiences of Dominicans and emphasize the importance of implementing culturally competent health programming and care.

## Introduction

According to the World Health Organization, over 1 billion people are estimated to be living with hypertension, a preventable, but major cause of premature morbidity and mortality [1]. Because hypertension and associated health behaviors are influenced by health beliefs (i.e., culturally informed perceptions related to sources, consequences, and management of disease [2]), recognizing and understanding their role is critical to public health intervention efforts [3–4]. Globally, there remains a need to develop and implement culturally relevant health programs and deliver culturally congruent care. Such movement underscores the increasing relevance of health beliefs, especially in low-resourced communities (i.e., communities lacking infrastructure and social services) in the Dominican Republic (DR).

The DR is of particular interest as nearly 35% of Dominicans have hypertension. With prevalence rates escalating annually, the condition has become a concern of growing importance [5]. In many countries in Latin America, including the DR, health beliefs are influenced by a rich and complex socio-historic blend of several cultural groups, including aboriginal populations, enslaved Africans, and Spanish colonizers [6]. These beliefs and associated practices highlight respect, attention, and the presence of family members and influence Dominicans’ tendency to utilize both traditional folk remedies (e.g., herbal or other tonics or treatments prepared at home without medical supervision or prescription) and professional healthcare [7]. Relatedly, research has highlighted that Dominicans perceive physical, spiritual, and mystical factors to be associated with causing, preventing, and treating disease [8–10]. Such perceptions have been associated with seeking treatment from traditional healers and demonstrate the inextricable connection between culture and health beliefs [8].

Despite the research on general health beliefs, research on the health beliefs of Dominicans regarding hypertension is scarce and much less information is available on those in low resource communities. Given that health beliefs are associated with lifestyle choices, use of healthcare services, and medication adherence [11,12], the importance of identifying health beliefs of those in low resource communities is magnified as individuals in these communities often experience the greatest disparities in health [13]. Furthermore, providers’ understanding of patients’ health beliefs is a key component of compassionate care–which facilitates more effective patient provider communication and results in perceptions of higher quality care among patients [14]. Thus, examining hypertension-related health beliefs and knowledge of Dominicans in vulnerable social locations would prove useful in providing domestic and foreign health professionals and practitioners with information that can assist in identifying gaps between patients’ and providers’ understandings of hypertension while guiding them in improving care for this group. Given the need to address the growing issue of hypertension and related illnesses in the DR, the current study expands the limited body of work on health beliefs and culture by focusing specifically on a low-resourced Dominican community.

## Methods

The Institutional Review Board (IRB) at Virginia Commonwealth University approved this study (Approval Number: HM15221). Verbal consent was obtained from all participants. This study was part of a larger IRB approved health needs assessment study that sought to identify community needs and health beliefs related to chronic illnesses, specifically hypertension and diabetes. The needs assessment was conducted in an effort to improve the healthcare services being offered by a medical mission group.

### Participants

Participants were recruited during the summer of 2013 from a small community in Santo Domingo, DR’s densely populated capital city, where 3 of the country’s 10 million inhabitants reside [15]. About 23% of the population in the DR lives below the national poverty line. The current study was conducted in Esfuerzo del Paraíso of Santo Domingo Norte [15]. The community, where participants were recruited, is situated on a river and is prone to frequent flooding (especially during the rainy season), which can make entering or leaving the community difficult. In addition, many residents do not have rapid or reliable access to food, healthcare, or social services.

To recruit, we employed purposive, convenience, and snowball sampling strategies. Participants were referred by word of mouth by interviewees, a medical mission doctor, and members of a Community Health Committee (CHC), a group of community residents selected by community members to promote health and serve as a point of contact for health initiatives. Referred individuals were primarily persons with hypertension. However, this was not a requirement for participation. To be included in the study, eligible individuals had to self-identify as a Dominican citizen, resident of the community in Santo Domingo (i.e., where the interviews took place), and be at least 18 years of age.

### Procedure

In this study, we operated within the interpretive paradigm, which maximizes subjectivity, places participants at the center of the research, and encourages researchers to use information gathered to produce meaning from the perspective of participants rather than simply conduct observations [16]. We conducted 18 semi-structured interviews with 20 participants. Two interviews included husband and wife dyads. Interviews took place inside and outside of participants’ homes and lasted between 20 and 40 minutes. Interviews were conducted in English to Spanish translation with an experienced medically trained interpreter and members of the CHC. The interview team consisted of an African American female interviewer with functional proficiency in Spanish (first author) and a Latino interpreter with five years of experience as a Spanish interpreter in healthcare settings (second author). Additionally, a CHC member and an observer, who composed detailed field notes, were present during each interview.

Interviews began with a review of confidentiality, followed by obtaining verbal consent for participation and permission to record the interview. Written consent was not obtained due to traditionally high rates of illiteracy in the community of interest. The verbal consent procedure was approved by the IRB and documented via a consent log. After asking demographic questions, participants were asked a series of questions to assess beliefs, knowledge, and experiences related to hypertension and diabetes. Questions relevant to the current study included: 1) What do you think/how do you feel about high blood pressure?; 2) What do you think provokes (or produces) high blood pressure?; 3) What can be done to control high blood pressure?; 4) Why do people get high blood pressure?; What impact, if any, does high blood pressure have on your life (daily activities like work, things to do, house chores, etc.)?; What can be done/what are you doing to control high blood pressure? What makes it difficult to control high blood pressure? What is the best way to treat high blood pressure? After all questions had been asked, the interviewer asked participants if they had additional comments or questions. Participants were then thanked for their participation. Interviews were conducted until data saturation (i.e., the point of data collection when gathering fresh data no longer sparks new insights) was achieved [17,18].

### Data analyses

Thematic analysis of data was based on qualitative research guidelines outlined in five basic phases: (1) become familiar with the data, (2) generate initial codes, (3) identify themes, (4) review/revise themes, and (5) define and name themes [19]. Five trained research team members, including the interviewer and the interpreter, transcribed English data collected on audio recorders. The interviewer and interpreter verified transcripts by checking for and editing translation inconsistencies. Afterwards, they reviewed all transcripts to become familiar with the data and generated a preliminary coding scheme, including 81 codes.

Guided by the recommendations of Braun and Clarke [19], each researcher used the preliminary coding scheme to independently assign codes to each unit (words, sentences, and/or phrases) of relevant data via NVivo 10, a qualitative data analysis software program. Data coding yielded 31 additional codes for a total of 112 codes. After the initial coding phase, the researchers merged similar codes, resolved coding discrepancies, and established inter-coder reliability at an agreement of 96%. From this process, 16 prevalent codes, those mentioned by at least 10 interviewees, emerged. Next, prevalent codes were grouped by similarity and relevance into themes. The following themes emerged from analyses: 1) Negative emotions are a primary cause of hypertension, 2) Medication is the best treatment but adherence is challenging, 3) Systemic barriers impede treatment access, 4) Hypertension negatively impacts mental and physical well-being, and 5) Lifestyle changes, relaxation, and social support help manage hypertension.

In sum, the data analytic approach was cyclical and involved continuous development of new codes and constant comparison of themes. This emergent design was essential to capturing the experiences, voices, and beliefs of respondents [17,20].

### Accounting for language and cultural differences

Several recommended strategies [21] were utilized to account for language and cultural differences. Principally, CHC members were present at each interview and served as cultural experts. They clarified questions and responses, addressed potential participant bias (i.e., the tendency for participants to respond to research questions in a socially desirable manner), and explained and contextualized reactions. Also, the interviewer encouraged elaboration on unclear or vague statements and used clarifying questions to ensure a valid understanding of responses [20]. To further account for language and cultural difference and personal biases, the interviewer and interpreter engaged in reflective practice via bracketing and taking field notes [17]. Before and after interviews, the interviewer and interpreter met to reflect, discuss, and compare personal perceptions. This helped the researchers become more familiar with cultural nuances and regionally specific vernacular. These processes allowed assumptions and predispositions to be acknowledged, explained, and monitored [21].

### Enhancing validity: Member checking

Member checking, or respondent validation, was conducted to validate the veracity of information gathered and to further ensure an accurate representation of data after translation and analysis. Member checking was conducted via brief discussions with original participants (*n* = 10; 53%) and CHC members (*n* = 5). Although CHC members were not interviewed, they helped to clarify misunderstandings and elaborate on native colloquialisms. During the follow up interviews, individuals received a copy of the study’s themes and subthemes (printed in Spanish) and the form was reviewed with participants to ensure understanding. After having the opportunity to review and discuss the document, participants were asked if any information should be omitted or added to the findings. Member checking participants did not receive any additional incentives. Results of the member checking are presented after results of the thematic analysis.

## Results and discussion

### Participant characteristics

In total we interviewed 20 Dominican men (*n* = 8; 40%) and women (*n* = 12; 60%) from a low resource community in Santo Domingo, DR. Participants ranged in age from 32 to 92 (*M* = 53, *SD* = 13.86) and half were employed (*n* = 10; 50%). Fifty five percent of participants (*n* = 11) were living with their partner, 30% (*n* = 6) were married, 10% (*n* = 2) were single/never married, and 5% (*n* = 1) identified as a widow. The majority identified as nonsmokers (*n* = 17; 85%). Fifteen participants (75%) reported having hypertension.

### Themes

#### Negative emotions are a primary cause of hypertension

Although participants discussed heredity, fatigue, and pain as precursors to hypertension, the most commonly referenced causes of hypertension were forms of psychological distress and associated lifestyle habits, mentioned by 47% (*n* = 9) and 32% (*n* = 6) of participants respectively. Psychological distress included the experience of anger, frustration, anxiety, and stress. For the individuals interviewed, stress was characterized by various daily hassles and the experience of being overwhelmed with life. One woman shared her experience with how she believed stress contributed to having hypertension, “My children, they make me have bad blood… I have so many things to do… and then I’m fighting with the children. I’m worried! I’m the man and the woman here, for everything… So it’s the stress…” (55 year-old woman). “Bad blood” was described by participants as having difficult interpersonal interactions that caused internal anger, stress, or frustration. For example, the participant who referenced her children appeared to be burdened with responsibilities and stressful interpersonal interactions and believed that these experiences directly contributed to her having high blood pressure. In like manner, a 62 year-old woman explained, “So many times… people have these diseases that … they weren’t meant to have …It’s just from worry, from things that happened in the home.”

Further explaining the perceived impact of psychological distress on hypertension, one woman described how her husband’s blood pressure went up whenever he experienced anger or frustration. “It attacks him, his high blood pressure, when something is paining him, when he is thinking about something, and when he gets angry, when he is speaking. His blood pressure goes up…” (57 year-old woman). Specifically, some participants believed that anger and frustration could contribute to the heating up of blood, which could cause blood pressure to rise and lead to adverse health outcomes. “When you fight your blood can heat up and [the blood pressure] goes up and then you can have a heart attack” (53 year-old man).

Relatedly, other participants (*n* = 4; 21%) believed the heat was related to having high blood pressure. “At times it goes up, especially when it’s really hot… in the wooded areas where there is a lot of shade I feel great but when the heat goes up I feel bad, I don’t feel well” (70 year-old man). Another participant commented on how the heat could indirectly cause a person to have high blood pressure by preventing health promoting behaviors known to reduce hypertension such as exercise. “It has impacts because sometimes you want to do exercise but you can’t.” (35 year-old woman).

### Medication is the best treatment but adherence is challenging

Most participants (*n* = 15; 79%) believed medication, commonly referred to as “treatment,” was useful in controlling or treating hypertension. Despite more participants using lifestyle changes versus medication (11 compared to 9) to control their hypertension, overall most participants believed that medication was the best treatment–highlighting a discrepancy between beliefs about the best treatment (medication) and the practices engaged in to control hypertension. Many viewed medications as superior to lifestyle changes as the “medication from the physicians… [is]…the most effective” (64 year-old woman). A 55 year-old woman stated, “for me that’s the best…Those are the best, it’s been four years, taking pills and medications.” As stated simply by a 47 year-old man, “the medications, those are better.” Medications were also viewed as more effective than home remedies such as herbal teas or plant based treatments (e.g., consuming garlic cloves).

When asked more specifically about home remedies as a treatment option for hypertension, some participants (*n* = 6; 32%) expressed feelings of aversion and distrust. “Home remedies, those aren’t the solutions because if they were the solutions then the problems would already be resolved. So you go to the physician” (41 year-old caretaker of hypertensive brother-in-law). Relatedly, participants also expressed a desire for healthcare providers, domestic and foreign, to provide educational sessions about hypertension so that community members could be aware of disease causes as well as how to best care for themselves when they do not have access to provider or medication.

Additionally, participants acknowledged the importance of adhering to medications. A 92 year-old man stated, “You have to take your medications and know how to follow through with them.” Similarly, a 70 year-old man commented, “take the medication because that’s the essential piece and go to the doctor take the medications as prescribed.” Despite being aware of the importance of adherence, participants acknowledged that they were not always adherent and discussed associated challenges. A 35-year old woman with gestational hypertension explained, “Sometimes people will take the treatment for 2 days and then they'll stop.”

When asked why individuals do not adhere to medication regimens, a 32 year-old woman shared her experience.

The two times they have prescribed me the medications and both times I have given them away … I give them to someone else who needs them more than me… My mother suffers from [high] blood pressure, since she’s not able to go to the physician … I gave it to her.

Other participants discussed similar instances of giving away of medications to loved ones as a reason for non-adherence, while others highlighted more systematic barriers to engaging in treatment and care for hypertension.

### Systemic barriers impede treatment access

Seventeen of the 19 individuals (89%) interviewed about hypertension, referenced barriers to treatment of hypertension. Twelve participants (63%) indicated difficulty in accessing healthcare services or treatments, with most (*n* = 11; 58%) citing poverty and challenges with money as an obstacle. A 53 year-old man explained, “Here, the money, we don't have much of that. Sometimes you have it and sometimes you don't, you know. We’re poor in this neighborhood; sometimes you don't have enough money to buy what [you] need, [like] the medications.” Likewise, a 35 old year woman mentioned that money was a barrier to obtaining medications, highlighting that “the medications are so expensive.”

A 55-year-old woman expressed frustration and worry that her husband, who also has hypertension and experienced a stroke, might have health complications due to not having enough money to purchase medication. “So the thing is …the money… I need those medications and … I don’t have enough money to buy the medication and when he doesn’t have that medication he has those attacks.” Providing context for this challenge, few participants mentioned that the lack of stable employment contributed to diminished access to treatment in the form of less income and loss of employer-based health insurance.

#### Challenges in the healthcare system

Five participants (26%) discussed barriers to treatment associated directly with the healthcare system. These issues included apathy from providers, overcrowded hospitals, and lack of available medications. One participant expressed annoyance as she imitated the lack of concern for patients displayed among healthcare professionals. “A lot of times and you go see the physicians and they are like ‘Ha Ha Ha.’ And you just wait [and after waiting so long] that’s when you go to the emergency room” (35-year-old woman). A 53 year-old man elaborated on difficulties experienced with the healthcare system, “so we have a public health system where we can go and they can give us medication only if it’s available because when there is none available then they can’t give [it to] us.”

Additionally, several participants (*n* = 4; 21%) mentioned the healthcare system’s neglect of individuals with physical impairments, discussing how mobility challenges are a barrier to making appointments and accessing treatment. A 49-year old woman with a hypertensive husband stated that “if [people] have the ability to go, they can get treatment but there are some people who can't really leave home because they have other sicknesses that won't allow them to.” In sum, barriers to treatment of hypertension referenced by interviewees included money, an unstable healthcare system, and physical difficulties that limit mobility and subsequently access to healthcare and treatment.

### Hypertension negatively impacts mental and physical well being

Nearly 75% of interviewees (*n* = 13) discussed the impact of hypertension on their life. Conversely, four (21%) individuals stated that hypertension had no impact on their life. About 32% of participants (*n* = 6) reported an impact on their physical health, such that the condition altered their body, making it difficult to sleep and leading to fatigue. Hence, they felt unable to go outside in the sun, work, complete chores, or do strenuous activities. One participant claimed that hypertension is caused and exacerbated by heat such that “you can’t handle things that are hot … so you need fresh things…” (41-year-old man). A 64-year-old woman described the impact of hypertension on her everyday life, highlighting the physical symptoms:

… for example…the house chores—I do them part by part because … in terms of … your body just breaking down, you can do all of it and you’re fine, but then you feel really exhausted… so sometimes I have to just sit down and calm myself down so that I can get back.

Relatedly, a 49 year-old man shared that “I would love to do everything. Before I could do everything and keep up with the family. Now I can’t do anything, I can’t work. I’m not able to meet the needs at home. I want to but I can’t.” A 47-year-old man suggested that “you just have to take it, the little transition, you have to take it softly, step by step [so you don’t get] too aggressive or… too agitated.”

Five participants (26%) expressed changes in mental health from hypertension. Mostly, interviewees reported feeling sad and overwhelmed, but also highlighted “needing to take things slow” or “relax.” A 64 year-old woman reported

… not feeling very well. It’s like if you were lying down and you had been sick for three days but nothing’s really wrong with you and it’s like you just feel, it’s like your face is down. It’s something that’s silent; the other thing is… it doesn’t hurt… [Like when] I’m walking and doing my house chores and [my neighbor] for example she asks me how do you feel and I don’t feel anything but she says your face looks … downward.

This woman believed she experienced melancholy because of having hypertension. For her, and others, the condition manifested physically and mentally.

### Lifestyle changes, relaxation, and social support help manage hypertension

Several other participants (*n* = 10; 53%) discussed the benefits of relaxation on hypertension. Interviewees expressed that keeping calm and “taking life as it comes” were one of the best ways to manage hypertension. “The best treatment for conditions like what I have is to be calm, to not have bad blood, not to get mad, not be in fights … That’s what I do, that’s the best treatment” (53 year-old man). “Relaxing in terms of lifestyle… can also treat it” (41 year-old woman). Other participants highlighted the significance of maintaining good psychological health, stating that it is important to “not have worries” (32 year-old woman) and have “control in life …and be calm” (70 year-old man). A few participants that highlighted “relaxation” as a form of treatment stated that doing so would be best in combination with taking medication and making better dietary and exercise choices. About 58% (*n* = 11) of participants believed that a lifestyle change, including dietary changes, increased exercise, and relaxation, could help treat hypertension. Specifically, participants stated that it is important “to have a personal step by step vigorous activity [plan]” (41 year-old caretaker of hypertensive brother-in-law) and “not to eat a lot of fat” (53 year-old man).

Utilizing networks of social support was discussed as another strategy for overcoming challenges associated with having and treating hypertension. Some interviewees relied on family and community members for assistance. One participant said,

… I don't have health insurance. My family, they help me when they can, at times my neighbors, my children- they’re helping me now since … [my employer is] not paying. When they start paying me again, I’ll have health insurance, but for now it’s my friends, neighbors, family members, my children (53 year-old man).

However, more than a quarter of those interviewed (*n* = 6) mentioned that assistance from others in the community, including family members, was not always available when they needed it. A 60 year-old man explained, “I don’t have any medication… my children they’re too busy.”

### Validation of findings via member checking

Member checking was conducted via brief discussions with half of the original participants (*n* = 10; 53%) and CHC members. Interviewees mostly reinforced perceived causes of hypertension, non-medical methods of hypertension management, and experiences with difficulty accessing healthcare services. However, one 38 year-old man did not support the contention that sun exposure and strenuous work caused hypertension. Spicy food and lack of food, and lack of medications for hypertension control were cited as causative factors of hypertension flare ups, mentioned by a 41 year-old woman and a 47-year old male. Individuals supported findings that highlighted drinking cool liquids and living a tranquil lifestyle as methods that help ameliorate high blood pressure. When discussing barriers to healthcare services, many agreed with responses from original interviews, stating that a lack of money prevented the purchase of medications and food and that obtaining medications was difficult due to institutional barriers. Two participants further explained that when people go to the hospital, they are sometimes sent home due to lack of medical personnel or medications. A CHC member also endorsed these views and added that such difficulty can force people to turn to home remedies.

## Discussion

The purpose of our study was to examine beliefs and knowledge related to hypertension in the DR. Findings demonstrated that participants understood the medical importance of treating and controlling hypertension and were aware of long-term physical and mental risks associated with the condition. Similar to participants in other studies, interviewees demonstrated a colloquial understanding of biological, lifestyle, and psychological predictors of hypertension, including heredity [22], diet and exercise [23], anger or “bad blood” [24], and occupational stress [25]. In addition to confirming findings in previous research, this study adds new findings to the literature regarding hypertension-related health beliefs and practices of Dominicans from a low resource community. For example, participants in the current study identified the sun and/or heat as a cause of hypertension, a belief substantiated by previous research. Although there is not a wealth of research in this area, there is a small body of literature that associates higher ambient temperatures with increased risk of cardiovascular events [26–28]. Thus, it is indeed possible that, as stated by a participant, blood pressure “… goes up, especially when it’s really hot.”

Most noted that medication plays an important role in controlling hypertension. Participants also highlighted the importance of adherence to medication and lifestyle changes such as increased exercise and healthier eating habits. Although participants were readily able to identify effective methods used to treat hypertension, they also identified significant barriers to obtaining or engaging in consistent treatment. Results revealed the experience of numerous personal and systematic barriers to care. Of these, difficulty accessing healthcare services was highlighted as a major issue, as was lack of stable employment (which affected both income and health insurance status). Both participant financial situations and barriers within the healthcare system (e.g., lack of available medications) influenced access to healthcare. Barriers to care and treatment adherence identified in the current study are similar to those identified by other qualitative studies conducted in low-middle income countries [29,30].

However, in addition to highlighting barriers to obtaining care, participants identified medication sharing as a personal barrier to treatment adherence, another unique finding of the current study. Specifically, participants explained the process of giving their medication to loved ones in need and/or taking medication prescribed to a loved one. This practice may help explain poor rates of treatment adherence in the DR. For example, in a sample of 6,400 Dominicans, 75% of those with hypertension did not follow recommended treatment [31]. It is possible that as a manifestation of the collectivistic nature of many Dominicans, those taking hypertension medication are less adherent because they desire to improve the health of their loved ones by supplying them with their own prescribed medication. These finding also highlight the need for interventionists to incorporate socioeconomic programs for patients who are unemployed and have family members depending on them for assistance. Additionally, the current study also identified supportive social networks as facilitators of treatment for hypertension, similar to previous research [32]. This is another important factor for public health interventionists to consider for future programs.

### Implications

Findings of the current study highlight the need to address barriers to care and treatment adherence at macro and micro levels. While several organizations have developed plans to create multi-stakeholder partnerships between governments, care providers, and patients to reduce the burden of non-communicable diseases, many health programs in the DR have not been reviewed or implemented due to a lack of human resources and inadequate funding and organization [33]. Results of the current study reaffirm the need to develop and adequately fund culturally appropriate hypertension prevention, management, and treatment programs at individual, community, and governmental levels. By taking the population’s health beliefs and related cultural norms into account, the impact of such community level interventions and action plans could be maximized [34].

Results of this study can also inform the work of foreign and domestic health professionals that provide hypertension-related care in the DR. Due to the expansion of university and faith-based service projects, rising numbers of short-term health missions have expanded healthcare access to global communities in need [35]. However, concerns have been raised regarding ethical issues and social, physical, and psychological damage caused by mission groups (e.g., unsafe medical practices, allowing individuals without proper credentials to treat patients, and inaccurate assumptions about patients along with lack of familiarity with language and culture of the host country that result in unintended offenses and/or inappropriate and unsustainable treatment) [36–39]. To avoid and overcome these challenges, it is important to become familiar with the culture, beliefs, practices, and contextual realities of the population of interest.

Beyond health missions, one can then use results from the current study to inform and strengthen approaches to other domestic and foreign prevention and treatment efforts [7]. For example, providers might relate community perceptions of disease causes (e.g., “bad blood”) to more medically accepted causes of illness (e.g., stress) and provide patients with culturally relevant strategies (e.g., “taking life as it comes” or stress management) for coping with and managing environmental, psychosocial, and biological causes of hypertension. In addition, patient-provider communication can be improved when providers perceive and understand patient’s health beliefs, which may result in improved patient decision-making and experiences of higher quality care among patients [14]. Establishing culturally competent health practices can also build rapport by demonstrating recognition and respect for the patient’s culture [40]. To the extent that health beliefs directly reflect cultural practices, identifying and understanding the impact of culturally specific health beliefs are vital to providing culturally competent healthcare domestically and abroad.

### Limitations

Although these findings are valuable, there are limitations. One limitation is the potential for participants to have misunderstood interview questions. It is possible that translation rendered researcher questions and participant responses unclear. Researchers attempted to minimize this possibility by utilizing a trained and certified medical Spanish interpreter, and asking CHC members to clarify questions and responses for researchers and participants. Further, researchers utilized member checking to ensure accurate understanding and representation of participant responses. Another limitation is the use of participant referral was utilized as a sampling strategy, which may have resulted in selection bias. Despite these limitations, results contribute to the growing body of research on health beliefs among Latin Americans and Dominicans in particular.

## Conclusions

Results of the current study emphasize the necessity and challenge of conducting culturally relevant health related programming and connecting individuals to reliable healthcare services in low-middle income countries. Health beliefs of participants revealed a myriad of personal, communal, and institutional challenges to obtaining care. All of which should be considered by health professionals interested in delivering culturally competent healthcare services abroad. Participants in this study were aware of the risk of uncontrolled hypertension and recognized the value of ongoing medical care. Such findings further support the importance of developing local and national action plans for diagnosing and treating hypertension, and moreover, lend themselves to guiding the work of both foreign and domestic health professional organizations.

## Supporting information

S1 File (DOCX)
