# Supplementary material for: Conocimientos de la hipertensión: Health beliefs about hypertension in an under-resourced community in the Dominican Republic
Source: PLoS One. 2020 Jun 23;15(6):e0235088. doi: 10.1371/journal.pone.0235088 (PMC7310720; doi:10.1371/journal.pone.0235088)
Supplement: S1 File — (DOCX) [file pone.0235088.s001.docx]

**Interview Questions**

**Community Members**

1. Have you or anyone in your household or family been diagnosed with diabetes or high blood pressure?
2. How did you/they learn you/they had this illness? How was the diagnosis made?
3. What do you know about high blood pressure?
4. What do you know about diabetes?
5. What causes these illnesses?
6. What impact, if any, do these illnesses have on daily life/activities?
7. What can be done/what are you/they doing to treat these illnesses?
8. What prevents you or others in the community from treating these chronic illnesses?
9. How can access to treatment be improved?
10. What are some of the resources to which you/they have access that help you/them take good care of your/their chronic illnesses?
11. What can the following groups do that will allow you/them to take better care of your/their chronic diseases?

a. Individual patients?

b. Dominican health care providers?

c. US healthcare providers?

d. The community?

1. What is the best way to treat chronic diseases like blood pressure or diabetes? (i.e., medicines, lifestyle changes such as exercise or diet changes, home remedies or traditional treatments)
